# On the Anatomy and Diseases of the Urinary and Sexual Organs

**Published:** 1845-06

**Authors:** 


Art. VI.—*On the Anatomy and Diseases of the Urinary and Sexual Organs.* Containing the Anatomy of the Bladder and of the Urethra, and the treatment of the Obstructions to which these passages are liable. By G. J. Guthrie, F.R.S., Surgeon to the Westminster Hospital, and to the Royal Westminster Opthalmic Hospital, &c., &c. From the Third London Edition. Philadelphia: Lea & Blanchard. 1845. 8vo. pp. 150.

This monograph describes the nature and treatment of a class of affections which often prove exceedingly embarrassing to the young surgeon. The author professes to have aimed at rendering the directions for the management of each particular complaint so plain, "that any one of even moderate capacity may understand and practice them." He has divided his work into two parts, of which the thin volume before us is the first; "the second will follow with the least possible delay."

The part published treats of the following subjects: the structure of the bladder and of the urethra, the formation of spasmodic and permanent stricture, and the means of its cure, the treatment of impassable stricture, the suppression and retention of urine, and irritation of the membranous and prostatic parts of the urethra.

The first chapter opens with the following remarks expressed in a style so chaste and elegant that we take great pleasure in quoting them:

"The continual calls on the bladder in both sexes, from the commencement to the termination of life, under circumstances which are frequently foreign to their natural state; and the double function which the uretra has to perform in man, during a long period of what is often an artificial mode of living, tend constantly to their derangement; and whilst, like every other part of the body, they contain within them the seeds of their decay, which would sooner or later develop themselves in the ordinary course of nature, they are often caused to germinate at a much earlier period, by the irregularities and vicious propensities of man himself.

"The diseases to which these parts are liable would be rarely experienced until the middle period of life was passed, if it were not for the indulgencies and irregularities which usually prevail in a highly civilized community, and more commonly give rise to them. They are not, however, the less sensibly felt, because they are sometimes merited. They are always, when severe, the source of great anxiety of mind and distress of body, leading gradually to premature decay; and terminating not unfrequently in a protracted and painful dissolution. At an early period, surgery can afford great and important relief; it can as often prevent further mischief as it can cure that which exists, and is a source of no less satisfaction to the surgeon than of delight and happiness to the patient. I know of no diseases for the cure of which the gratitude of one man to another is more often and more cordially expressed.

We shall not follow the author in his description of the anatomy of the urinary organs, but give from the chapter on the structure of the bladder the subjoined instructive observations:

"When the bladder is contracted and empty, the urine passes readily into and gradually dilates it, until the desire for expulsion comes on and leads to its evacuation. A little more or a little less seems to have no influence in preventing the urine from finding its way in, the weight of the column descending from the kidney readily overbalancing to a certain point the resisting power of the coats of the bladder.

When the bladder is distended, it no longer yields easily, the ureter is pressed upon by the muscular wall in its passage through it, and the further entrance of urine is in a great measure prevented. If the obstruction be long continued, the ureter above this part is gradually dilated from the size of a crow quill to that of a man's thumb, and even larger; the pelvis of the kidney increases in size, a low or chronic inflammation is induced, the secretory organs are pressed upon and partially removed, so that the kidney may become at last an almost empty bag separated by partitions, indicating only the former existence of its lobes. A total suppression of the secretion may, under such circumstances, take place at any time. The most remarkable example of the kind which has come under my observation, occurred in the case of a lady who suffered from a cancer of the uterus; the disease after a time extended towards the ureters, which at last were embraced and pressed upon by it as they entered the bladder. The lady, as this took place, began to suffer more from derangement of her urinary apparatus; the bladder was found ultimately, on passing the catheter, to contain little or no water; she fell into a state of low fever, became paralytic, afterwards comatose, and died. On examination, the orifices of the ureters were found in a sound state, although the ureters were impervious at the part where they were grasped by the diseased structure; above this they were greatly enlarged, and the kidneys were much diseased and sacculated. The peculiar manner in which the ureters enter into the bladder is, then, an admirable provision of nature for the purpose of preventing too great a distention of the bladder rather than of the ureters, for nature can accommodate herself for several days to a complete suppression of the secretion of urine, and for a very long time to a partial secretion of it."

The second chapter considers the structure of the urethra and the instruments used in treating its complaints. It contains very full directions with which the practitioner ought to be familiar when proceeding to correct disorders of this passage. Speaking of false passages in the urethra the author says:

"Dr. Civiale, who saw, when in London, the care bestowed in the preservation of specimens of diseases of the bladder and urethra, in which false passages had been made, and which care is not taken in Paris, where they are rarely preserved, and where they are consequently not seen, concludes, in his 'Memoire sur l'Anatomie Pathologique des Retricissemens de l'Uretre,' that these evils have arisen in a great measure from the faulty construction of our catheters and sounds, which he considers to be too much bent. In London, we are apt to think that false passages occur more frequently abroad than at home, from the circumstance of their instruments being too conical and too straight."

The chapter on strictures is highly instructive. This affection may be spasmodic or permanent. The former, from the following remarks of our author, would appear to be a rare occurrence:

"The only case of what may be called pure spasmodic action which has come under my observation, occurred in a gentleman, who came to my house twice in the course of several years, declaring he could not make his water, and desiring to have a catheter passed; which was each time done without the least difficulty. The first time he came he was aware of his situation; said it arose from anxiety of mind relating to family affairs, and that the passage of the instrument would immediately and effectually relieve him. If there were an obstacle, and I was by no means certain of there being any beyond a hesitation, it was at the commencement of the membranous part of the urethra, and arising, I suppose, from a spasmodic contraction of the compressor urethræ muscle. As this gentleman suffered no kind of inconvenience at any other time, I am induced to believe that there was no particular irritation in the urethra, and that it was, as the cause is unknown, what may be called accidental spasmodic. I have heard of a lawyer, but I do not recollect where I heard it, who often suffered in this way when engaged long in court in a difficult case, and who was always relieved in a similar manner; but here I should say it is more than probable the individual was laboring under some slight permanent irritation in the urethra, or that it was at least in an excitable state at some one part near the bulb. A healthy man suffering from anxiety and alarm, often feels a desire to pass his water, which he cannot at all times restrain, and it flows whether he will or not; but if he has the power of restraining it for hours, then, indeed, the powerful contraction of the compressor urethræ may bring on irritation in the part, and spasm of the muscle; but this is the result of its own irregular and long continued action, inducing irritation as the first step to inflammation, and is of exceedingly rare occurrence; still it is not an instance of pure spasm, like the case I have related, in which the incapability was preceded by no uneasiness until the attempt at micturition was made."

The fourth chapter treats of symptoms of stricture, concerning which the author makes the following impressive remarks:

"The symptoms of stricture, like those of most other diseases not immediately affecting life, are often as slow in their progress, and as insidious in their nature, as they are appalling in their results. They are rarely present until after the age of puberty; are common from that period until after five and forty years of age; and seldom occur as the consequences of stricture afterwards: so that when an elderly man complains of symptoms of this kind for the first time, they usually indicate derangement of the prostate, the bladder, or the kidneys. I have a gentleman, advanced in life, now under my care, who sent for me for a retention of urine, declaring he had no stricture; yet he admitted that his urine for 30 years had passed in a very small stream, and that he had had occasionally a stoppage of water. This was clearly a complaint of the urethra of long standing; and, on examination, I found a stricture at five inches and a half, which would not admit, for several days, the smallest bougie."

After this follow directions for examining the urethra, and then remarks on the cure of the malady by dilatation. The work must be consulted for information on these points, since a compression of the details of practice into the limits at our disposal would be utterly impossible.

In the same chapter the mode of destroying strictures by caustic is described at length, and hemorrhage, and the division of a stricture are also considered.

The following chapter is taken up with the treatment of impassable stricture, of which the author says:

"When a stricture is impassable by the bougie, but is permeable by the urine, although it flows with difficulty, and there is no urgent necessity for the immediate removal of the obstruction, two different modes of proceeding may be adopted for its cure; one by a long-continued and equable pressure made on the face of the stricture by a pliable hollow gum-elastic bougie, with which I have usually succeeded in overcoming the obstruction when not of any great extent; and of effecting a passage into the bladder, without giving rise to the alarm and anxiety which more vigorous measures sometimes occasion. The other, by steady pressure made for a short time at intervals with a solid instrument, until the obstruction is gradually overcome."

Regarding the mode of obviating the obstruction he has the following:

"The best dilating material is a pliable hollow gum-elastic bougie, of a medium size, and perfectly smooth, and tolerably round at the point, so that it may give as little uneasiness as possible. This instrument is to be fixed in the urethra in the same way as a gum-elastic catheter is fixed in the bladder; it should project about one inch beyond the orifice of the urethra, and rather less than more. The point should press against or rest upon the stricture with the greatest possible gentleness, so that it may not give rise to inflammation or ulceration, and yet should press just so much as to cause absorption. It is an admitted point in the animal economy, that new-formed parts, whether laid down in reparation or in disease, do not resist a stimulus in the same manner as parts of original formation. They are in fact removed by the action of the absorbents under the application of a stimulus, which has little or no influence on parts which have undergone no change, and are coeval with the existence of the individual. The pressure made by the point of the bougie should therefore be nicely regulated, so that it may do this and no more. The patient readily learns what is wanted, and as he can feel when the surgeon cannot, he soon understands how to manage the bougie himself, and can take it out, wash it, change it, or replace it, as he pleases. If he should be a very restless, fretful, or naturally irritable man, it may prevent sleep or prove inconvenient, in either of which cases it may be removed for two or three hours, at the pleasure of the individual, whose private affairs may otherwise render this indulgence necessary. If any further irritation should take place, it ought to be subdued by warm fomentations, by opiates, and perhaps by the application of a few leeches. There are few cases which require anything more, provided the patient will be perfectly quiet, live moderately, and preserve the recumbent position, until the irritation has subsided."

On this topic, as on all the points discussed, Mr. Guthrie shows himself the experienced surgeon, and illustrates his views with striking and instructive cases. It is seldom indeed, that we meet with a work evincing so much of the scholar and the man of practice. One is at a loss which most to admire in it, its elegant style, or its practical usefulness.

The remaining chapters treat of suppression and retention of urine, and irritation of the membranous and prostatic parts of the urethra, but as we find it impracticable to present any thing like an analysis of their contents in the few pages we are permitted to occupy with this notice, we take leave of the volume, earnestly recommeding it to the careful study of the young surgeon. The publishers have conferred a favor on the American profession by bringing out a work of so much merit.

